# Supplementary material for: Urinary Metabolomic Differentiation of Infants Fed on Human Breastmilk and Formulated Milk
Source: Metabolites. 2024 Feb 16;14(2):128. doi: 10.3390/metabo14020128 (PMC10890188; doi:10.3390/metabo14020128)
Supplement: Supplementary file 1 [file metabolites-14-00128-s001.zip › metabolites-2756135-supplementary.pdf]

Supplementary data

Title: Urinary Metabolomic Differentiation of Infants Fed on Human Breastmilk and Formulated Milk

Supplementary Table S1. Information about urine sample

Neonate #	Type of milk feeding	Sex	Gestational weeks	Day 1		Day 7		Day 14		Mothers		
				Weight (g)	Height (cm)	Weight (g)	Height (cm)	Weight (g)	Height (cm)	body weight	height	diseases
1	Formular milk	Male	33	2210	48	2120	45	2270	47	58	163	0
2	Formular milk	Male	34	1900	42	1830	43	2040	44	71	151	0
3	Formular milk	Female	34	2070	43	1980	44.5	2220	46.5	71	156	0
4	Formular milk	Male	33	2500	46	2410	48	2660	49	61	155	0
5	Formular milk	Female	34	2220	44.5	2130	45	2400	46.5	71	156	0
6	Formular milk	Male	33	1950	44	1740	44	1900	44.5	60	159	0
7	Formular milk	Male	33	2110	43	2000	45	2130	45.5	81	165.6	0
8	Formular milk	Male	33	1480	40	1440	41	1580	42	81	165.6	0
9	Formular milk	Male	34	1900	44	1800	44	1970	45	62	160	0
10	Formular milk	Male	34	1780	43	1700	44	1830	44.5	62	160	0
11	Formular milk	Male	34	1880	42	1730	43.5	1900	46	68	160	0
12	Formular milk	Male	34	2240	45	2080	46	2210	47	80	160	0
13	Formular milk	Male	34	2430	50	2160	46	2260	47	71.7	164.2	0
14	Formular	Female	34	3190	50	2840	49.5	3120	50.5	63.7	157	0

	milk											
15	Formular milk	Male	33	2390	44	2150	45	2248	46	96	156	0
16	Human milk	Female	34	2260	44	2080	43.5	2260	44.5	57.4	165	0
17	Human milk	Female	33	1810	42	1600	43	1710	42	65	160	0
18	Human milk	Female	34	2590	47	2350	48.5	2440	50	97	168	0
19	Human milk	Female	34	2590	44	2390	47	2570	48	83	163	0
20	Human milk	Female	33	2370	45	2140	46	2320	47	94	167	0
21	Human milk	Male	33	1730	43	1540	41.5	1720	43	89.5	168	0
22	Human milk	Male	33	2050	42	1830	43	2060	44.5	89.5	168	0
23	Human milk	Male	34	1820	41.5	1720	41.5	1730	44	53	156	0
24	Human milk	Male	34	1900	42.5	1760	42	1830	44	53	156	0
25	Human milk	Female	32	2040	41.5	1900	41	2030	45	86	160	0
26	Human milk	Female	32	2080	43	1920	40.5	2080	44	86	160	0
27	Human milk	Female	34	2120	46.5	2000	43	2200	47	64	156	0
28	Human milk	Female	34	1970	43	1820	44	1890	46	65	156	0
29	Human milk	Male	34	2150	44	2120	44	2250	45	76.6	157.2	0
30	Human	Male	34	2300	46	2160	46	2250	47	76.6	157.2	0

	milk

Supplementary Table S2. Retention time of peaks from untargeted metabolomics

Peak #	Retention time (min)
1	12.344
2	12.855
3	14.393
4	14.673
5	15.78
6	16.19
7	16.398
8	17.548
9	18.087
10	18.334
11	18.59
12	18.735
13	19.028
14	19.325
15	20.454
16	20.799
17	20.95
18	22.589
19	23.1
20	23.381
21	23.753
22	24.297
23	24.61
24	24.715
25	24.94
26	25.425
27	25.657
28	26.031
29	26.14
30	26.266
31	26.589
32	27.04
33	28.22
34	29.226
35	29.331
36	29.425
37	29.694
38	29.935
39	30.155
40	30.361
41	30.473

42	30.657
43	30.97
44	31.105
45	31.259
46	31.395
47	31.664
48	31.8
49	32.136
50	32.473
51	32.715
52	32.995
53	33.282
54	33.46
55	33.681
56	33.911
57	34.065
58	34.172
59	34.285
60	34.507
61	34.702
62	34.825
63	34.993
64	35.215
65	35.482
66	35.605
67	35.728
68	36.053
69	36.231
70	36.49
71	36.599
72	36.747
73	37
74	37.167
75	37.479
76	37.599
77	37.959
78	38.198
79	38.38
80	38.469
81	38.791
82	38.935
83	39.207
84	39.331
85	39.519

86	39.71
87	39.82
88	39.989
89	40.136
90	40.49
91	40.714
92	40.873
93	41.05
94	41.275
95	41.422
96	41.545
97	41.699
98	41.891
99	42.277
100	42.37
101	43.069
102	43.24
103	43.582
104	43.769
105	43.945
106	44.049
107	44.17
108	44.319
109	44.753
110	44.953
111	45.111
112	46.195
113	46.417
114	47.238
115	47.707
116	47.83
117	48.023
118	48.148
119	48.401
120	48.803
121	48.94
122	49.32
123	49.588
124	49.813
125	50.095
126	50.322
127	50.584
128	50.845
129	51.344

130	51.58
131	51.76
132	52.183
133	52.335
134	52.697
135	52.894
136	53.181
137	53.423
138	53.656
139	54.06
140	54.298
141	54.415
142	54.551
143	55.015
144	55.21
145	55.39
146	55.6
147	55.769
148	56.129
149	56.365
150	56.581
151	56.8
152	56.938
153	57.16
154	57.265
155	57.415
156	57.67
157	58.078
158	58.18
159	59.057
160	59.364
161	59.576
162	59.68
163	59.83

Supplementary Table S3. Metabolites from targeted metabolomics

Compound	Class
1-Hexadecanol	alcohols
2-Aminoethanol	alcohols
Batyl alcohol	alcohols
diethanolamine	alcohols
docosanol	alcohols
eicosanol	alcohols
ethylene glycol	alcohols
Histidinol	alcohols
Metoprolol	alcohols
octacosanol	alcohols
Octadecanol	alcohols
Octopamine	alcohols
tetracosanol	alcohols
tetradecanol	alcohols
threoninol	alcohols
Triacontanol	alcohols
Triethanolamine	alcohols
1-Hexacosanol	alcohos
Allantoin	amides
N-benzylpalmitamide	Amides
Carnosine	amides
Creatinine	amides
Glycyl-Glycine	amides
Oleamide	amides
oxamic acid	amides
oxamide	amides
Pantothenic acid	amides
Urea	amides
3,4-Dihydroxybenzylamine	amines
Cadaverine	amines
Cystamine	amines
Fendiline	amines
Histamine	amines
homoglutamine	amines
Putrescine	amines
Spermidine	amines
Spermine	amines

Vanillylamine	amines
2-Aminoadipic acid	amino acids
2-Aminobutyric acid	amino acids
2-Aminoisobutyric acid	amino acids
2-Aminooctanoic acid	amino acids
2-Aminopimelic acid	amino acids
3-Aminoisobutyric acid	amino acids
3-Aminopropanoic acid	amino acids
3-Hydroxyanthranilic acid	amino acids
3-Hydroxy-kynurenine	amino acids
3-Methylcrotonylglycine	amino acids
3-Sulfinoalanine	amino acids
4-Aminobenzoic acid	amino acids
4-Aminobutyric acid	amino acids
4-Hydroxyproline	amino acids
5-Aminolevulinic acid	amino acids
5-Hydroxy-tryptophan	amino acids
Acetylglycine	amino acids
Alanine	amino acids
Anthranilic acid	amino acids
Arginine	amino acids
Asparagine	amino acids
Aspartic acid	amino acids
Citrulline	amino acids
Cystathionine	amino acids
Cysteic acid	amino acids
Cysteine	amino acids
Cystine	amino acids
Dimethylglycine	amino acids
Dopa	amino acids
Glutamic acid	amino acids
Glutamic acid 5-methylester	amino acids
Glutamine	amino acids
Glycine	amino acids
Histidine	amino acids
Homocysteine	amino acids
Homocystine	amino acids
Homoserine	amino acids
Isobutyrylglycine	amino acids
Isoleucine	amino acids

Isovalerylglycine	amino acids
Kynurenine	amino acids
Leucine	amino acids
Lysine	amino acids
Methionine	amino acids
Methionine sulfone	amino acids
methyl cysteine	amino acids
methyl serine	amino acids
N6-Acetyllysine	amino acids
N-Acetylaspartic acid	amino acids
N-Acetylglutamine	amino acids
N-Acetyl-Lysine	amino acids
N-Acetyl-Ornithine	amino acids
N-Acetylserine	amino acids
N-Acetyltyrosine	amino acids
N-Butyrylglycine	amino acids
Norleucine	amino acids
Norvaline	amino acids
O-Acetylserine	amino acids
O-Phospho-Serine	amino acids
Ornithine	amino acids
p-Aminohippuric acid	amino acids
Phenylalanine	amino acids
Proline	amino acids
Saccharopine	amino acids
Sarcosine	amino acids
S-Benzyl-Cysteine	amino acids
Serine	amino acids
Suberylglycine	amino acids
Threo-b-hydroxyaspartic acid	amino acids
Threonine	amino acids
Tiglylglycine	amino acids
Tryptophan	amino acids
Tyrosine	amino acids
Ureidopropionic acid	amino acids
Valine	amino acids
pyrrolnitrin	azoles
1,5-Anhydroglucitol	carbohydrates
1,6-Anhydroglucose	carbohydrates
2,3-Bisphosphoglyceric acid	carbohydrates

2-Deoxy-D-glucose	carbohydrates
6-Phosphogluconic acid	carbohydrates
Allose	carbohydrates
Arabinose	carbohydrates
Arabitol	carbohydrates
Ascorbic acid	carbohydrates
cellobiose	carbohydrates
Dihydroxyacetone	carbohydrates
Dihydroxyacetone phosphate	carbohydrates
D-Erythrose	carbohydrates
Erythrose 4-phosphate	carbohydrates
Erythrulose	carbohydrates
Fructose	carbohydrates
Fructose 1-phosphate	carbohydrates
Fructose 6-phosphate	carbohydrates
Fucose	carbohydrates
Galactitol	carbohydrates
D-Galactosamine	carbohydrates
Galactose	carbohydrates
Galacturonic acid	carbohydrates
Glucaric acid	carbohydrates
Gluconic acid	carbohydrates
D-Glucono-1,4-lactone	carbohydrates
D-Glucono-1,5-lactone	carbohydrates
Glucosamine	carbohydrates
Glucose	carbohydrates
Glucose 6-phosphate	carbohydrates
Glucuronic acid	carbohydrates
Glyceraldehyde	carbohydrates
D-Glyceraldehyde 3-phosphate	carbohydrates
Glyceric acid	carbohydrates
Glycerol	carbohydrates
Glycerol 2-phosphate	carbohydrates
Glycerol 3-phosphate	carbohydrates
Inositol	carbohydrates
Inositol phosphate	carbohydrates
Isomaltose	carbohydrates
Lactitol	carbohydrates
Lactose	carbohydrates
lactulose	carbohydrates

Lyxose	carbohydrates
Maltitol	carbohydrates
Maltose	carbohydrates
maltotriose	carbohydrates
Mannitol	carbohydrates
Mannose	carbohydrates
Mannose 6-phosphate	carbohydrates
melibiose	carbohydrates
meso-Erythritol	carbohydrates
N-Acetylmannosamine	carbohydrates
N-Acetylneuraminic acid	carbohydrates
palatinose	carbohydrates
Psicose	carbohydrates
raffinose	carbohydrates
Rhamnose	carbohydrates
Ribitol	carbohydrates
Ribonolactone	carbohydrates
Ribose 5-phosphate	carbohydrates
Ribose	carbohydrates
Ribulose 5-phosphate	carbohydrates
Ribulose	carbohydrates
saccharo-1,4-lactone	carbohydrates
Sedoheptulose 7-phosphate	carbohydrates
Sorbitol	carbohydrates
Sorbose	carbohydrates
Sucrose	carbohydrates
Tagatose	carbohydrates
Threitol	carbohydrates
Threonic acid	carbohydrates
Trehalose	carbohydrates
Trehalose 6-phosphate	carbohydrates
Turanose	carbohydrates
Xylitol	carbohydrates
Xylose	carbohydrates
Xylulose	carbohydrates
gluconic acid lactone	esters
Mevalonic lactone	esters
O-Phosphoethanolamine	esters
eicosaenoic acid	fatty acids
11-eicosenoic acid	fatty acids

5-Aminovaleric acid	fatty acids
Dihomo-gamma-linolenate	fatty acids
Acetoacetic acid	fatty acids
alpha-linolenic acid	fatty acids
arachidic acid	fatty acids
Arachidonic acid	fatty acids
behenic acid	fatty acids
Caproic acid	fatty acids
cis-10-heptadecenoic acid	fatty acids
cis-10-pentadecenoic acid	fatty acids
Decanoic acid	fatty acids
dihydrocapsiate	fatty acids
Docosahexaenoic acid	fatty acids
Docosapentaenoic acid	fatty acids
Eicosapentaenoic acid	fatty acids
Elaidic acid	fatty acids
heneicosanoic acid	fatty acids
heptadecanoic acid	fatty acids
Juniperic acid	fatty acids
lauric acid	fatty acids
Linoleic acid	fatty acids
Lipoic acid	fatty acids
Margaric acid	fatty acids
Methyl arachidonate	fatty acids
Myristic acid	fatty acids
myristoleic acid	fatty acids
nonadecanoic acid	fatty acids
Nonanoic acid	fatty acids
norhydrocapsiate	fatty acids
Octanoic acid	fatty acids
Oleic acid	fatty acids
Palmitic acid	fatty acids
Palmitoleic acid	fatty acids
pentadecanoic acid	fatty acids
Stearic acid	fatty acids
tetracosanoic acid	fatty acids
tricosanoic acid	fatty acids
tridecanoic acid	fatty acids
Valproic acid	fatty acids
Monostearin	glycerides

5-Methoxytryptamine	Indoles
indole-3-acetic acid	Indoles
Melatonin	Indoles
Tryptamine	Indoles
Hydroxylamine	inorganic compounds
2'-Deoxyuridine	nucleosides
5'-Methylthioadenosine	nucleosides
Cytidine	nucleosides
Thymidine	nucleosides
Thymidine monophosphate	nucleosides
Uridine	nucleosides
Uridine monophosphate	nucleosides
Xanthosine	nucleosides
Xanthosine monophosphate	nucleosides
Adenosine 3',5'-cyclic monophosphate	nucleotides
Adenosine monophosphate	nucleotides
2-Hydroxybutyric acid	organic acids
2-Hydroxyglutaric acid	organic acids
2-Hydroxyhippuric acid	organic acids
2-Hydroxyisobutyric acid	organic acids
2-Hydroxyisocaproic acid	organic acids
2-Hydroxyisovaleric acid	organic acids
2-Isopropylmalic acid	organic acids
2-Ketoadipic acid	organic acids
2-Ketobutyric acid	organic acids
2-Ketoglutaric acid	organic acids
2-Ketoisocaproic acid	organic acids
3-Methyl-2-oxobutanoic acid	organic acids
2-Methyl-3-hydroxybutyric acid	organic acids
2-Methylhippuric acid	organic acids
2-Phosphoglyceric acid	organic acids
3-Hydroxyvalproic acid	organic acids
5-Hydroxyvalproic acid	organic acids
2-Propylglutaric acid	organic acids
3-Aminopentanedioate	organic acids
5-Amino-5-deoxy-3-dehydroshikimate	organic acids
3-Hydroxy-3-methylglutaric acid	organic acids
3-Hydroxybutyric acid	organic acids
3-Hydroxyglutaric acid	organic acids
3-Hydroxyisobutyric acid	organic acids

3-Hydroxyisovaleric acid	organic acids
3-Hydroxyphenylacetic acid	organic acids
3-Hydroxypropionic acid	organic acids
3-Hydroxypyruvic acid	organic acids
3-Methoxy-4-hydroxybenzoic acid	organic acids
3-Methyl-2-oxovaleric acid	organic acids
3-Methyladipic acid	organic acids
3-Methylglutaric acid	organic acids
3-Phenyllactic acid	organic acids
3-Phosphoglyceric acid	organic acids
4-Hydroxybenzoic acid	organic acids
4-Hydroxyphenylacetic acid	organic acids
4-Hydroxyphenyllactic acid	organic acids
4-Hydroxyphenylpyruvic acid	organic acids
5-Dehydroquinic acid	organic acids
5-Hydroxymethyl-2-furoic acid	organic acids
5-Oxoproline	organic acids
Aconitic acid	organic acids
Adipic acid	organic acids
Azelaic acid	organic acids
Benzoic acid	organic acids
Biotin	organic acids
cinnamic acid	organic acids
Citramalic acid	organic acids
Citric acid	organic acids
coumaric acid	organic acids
L-Dihydroorotate	organic acids
Dodecanedioic acid	organic acids
Ethylmalonic acid	organic acids
ferulic acid	organic acids
Fumaric acid	organic acids
Glutaconic acid	organic acids
Glutaric acid	organic acids
Glycolic acid	organic acids
Glyoxylic acid	organic acids
Hexanoylglycine	organic acids
Hippuric acid	organic acids
Homogentisic acid	organic acids
Homovanillic acid	organic acids
Hypotaurine	organic acids

Isocitric acid	organic acids
Kynurenic acid	organic acids
Lactic acid	organic acids
Maleic acid	organic acids
Malic acid	organic acids
Malonic acid	organic acids
Mesaconic acid	organic acids
Methylmalonic acid	organic acids
Methylsuccinic acid	organic acids
Nicotinic acid	organic acids
Orotic acid	organic acids
Oxalacetic acid	organic acids
Oxalic acid	organic acids
Phenylacetic acid	organic acids
Phenylpyruvic acid	organic acids
Phosphoenolpyruvic acid	organic acids
Pimelic acid	organic acids
pipecolic acid	organic acids
Porphobilinogen	organic acids
Protocatechuic acid	organic acids
pyroglutamic acid	organic acids
Pyruvic acid	organic acids
quinic acid	organic acids
Ribonic acid	organic acids
Salicylic acid	organic acids
Sebacic acid	organic acids
Shikimic acid	organic acids
sinapic acid	organic acids
Suberic acid	organic acids
Succinic acid	organic acids
Succinylacetone	organic acids
Tartaric acid	organic acids
Taurine	organic acids
Thiodiglycolic acid	organic acids
Tropic acid	organic acids
Ureidosuccinic acid	organic acids
Urocanic acid	organic acids
Vanilmandelic acid	organic acids
Catechol	phenols
Coniferyl alcohol	phenols

Coniferyl aldehyde	phenols
Dopamine	phenols
Epinephrine	phenols
Hydroquinone	phenols
Norepinephrine	phenols
Pyrogallol	phenols
Tyramine	phenols
Phosphoric acid	phosphoric acids
7-Methylguanine	purines
Adenine	purines
Adenosine	purines
Guanine	purines
Guanosine	purines
Hypoxanthine	purines
Inosine	purines
Inosine monophosphate	purines
ParaXanthine	purines
Uric acid	purines
Xanthine	purines
3-hydroxypyridine	pyridines
4-hydroxypyridine	pyridines
Niacinamide	pyridines
Pyridoxal	pyridines
Pyridoxamine	pyridines
Pyridoxine	pyridines
Quinolinic acid	pyridines
Cytosine	pyrimidines
Dihydrouracil	pyrimidines
Thymine	pyrimidines
Uracil	pyrimidines
campesterol	steroids
Cholecalciferol	steroids
Cholestanol	steroids
Cholesterol	steroids
Sitosterol	steroids
Stigmasterol	steroids
lanosterol	terpenoids
Methoprene acid	terpenoids
alpha-tocopherol	tocopherols
delta-tocopherol	tocopherols
